# Supplementary material for: Vorinostat Improves Myotonic Dystrophy Type 1 Splicing Abnormalities in DM1 Muscle Cell Lines and Skeletal Muscle from a DM1 Mouse Model
Source: Int J Mol Sci. 2023 Feb 14;24(4):3794. doi: 10.3390/ijms24043794 (PMC9964082; doi:10.3390/ijms24043794)
Supplement: Supplementary file 1 [file ijms-24-03794-s001.zip › ijms-2131144-supplementary.pdf]

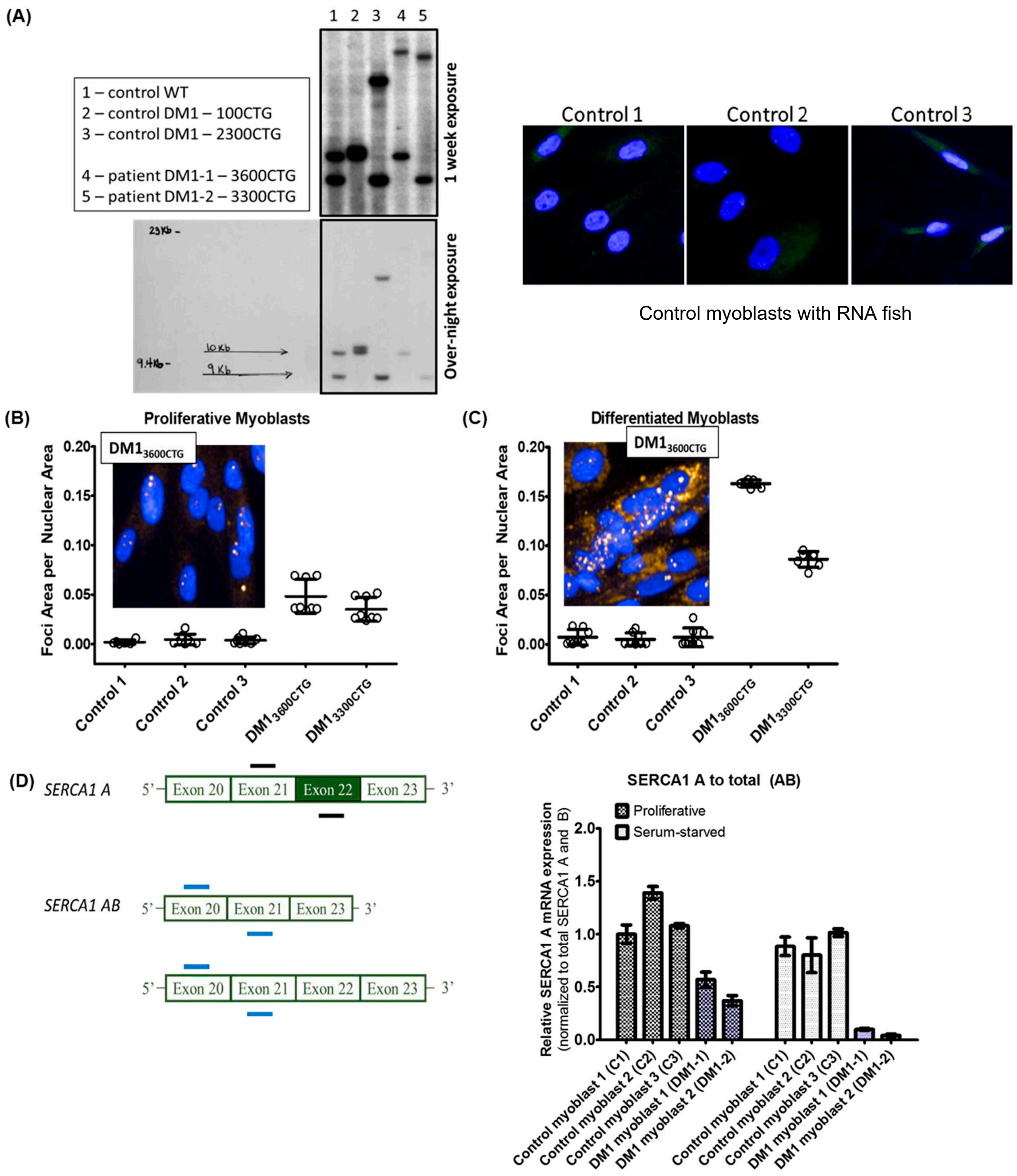

Fig. S1. DM1 myoblasts contained 3000+ CTG repeats, had intranuclear CUG-foci and had SERCA1 spliceopathy. (A) Southern blot analysis of genomic DNA extracted from two immortalized DM1 myoblasts (lane 4 and 5). Lane 1 contains DNA from an individual known to be void of the DM1 genotype (negative control); lanes 2 and 3 contain

DNA from individuals known to have DM1 with pre-established repeat sizes (positive control). Lanes 1-3 serve as representative controls used for diagnostic purposes at the CHEO genetic clinic. (B-C) RNA FISH for the analysis of intranuclear CUG-foci. Cells were grown and/or differentiated for 7 days in a 384-well plate (n=7) and fixed with 4% PFA. DNA was stained with Hoechst and CUG RNA foci were probed by Alexa555-$(CAG)_{10}$ oligonucleotide probe. Average foci area per nuclear area is presented from (B) proliferative myoblasts growing in Ham's F14 complete growth media containing 30% FBS or (C) differentiated myoblasts serum-starved in Ham's F14 growth media supplemented with 1% horse-serum. (D) RT-qPCR analysis of SERCA1 spliceopathy in proliferative and differentiated myoblasts. DM1-1 cells are $DM1_{3600CTG}$ and DM1-2 cells are $DM1_{3300CTG}$.

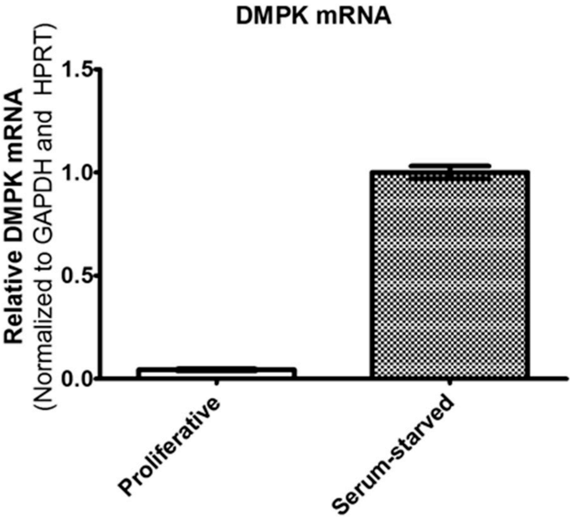

Fig. S2. DMPK mRNA expression is greater in serum-starved (differentiated) than proliferative $DM1_{3600CTG}$ immortalized myoblasts. Cells were grown in complete media (proliferative) or additionally serum-starved in differentiation media for 7, RNA was extracted and converted to cDNA, and DMPK mRNA was quantified using qPCR to compare expression between proliferative and serum-starved $DM1_{3600CTG}$ myoblast, normalized to GAPDH and HPRT.

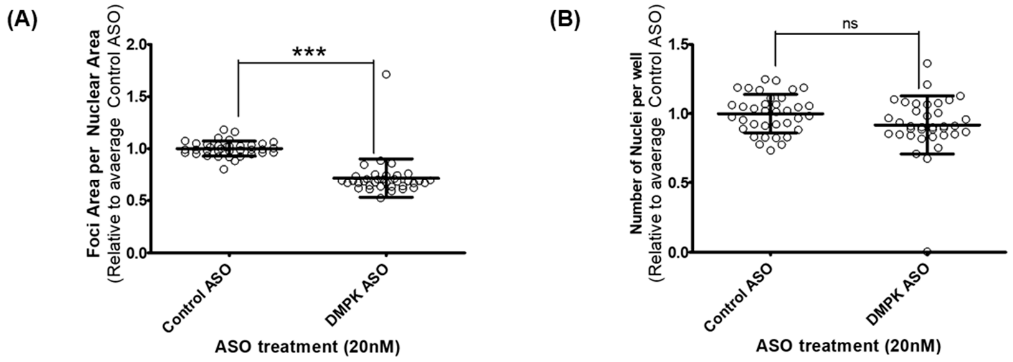

Fig. S3. DMPK ASO reduced foci by 30% in differentiated $DM1_{3600CTG}$ myoblasts. $DM1_{3600CTG}$ myoblasts were serum-starved in 384-well plates for 7 days and treated with 20 nM of control or DMPK ASO (ISIS486178) for 24 h (n=9, 6-wells per plate). Post-treatment, cells were fixed with 4% PFA, DNA was stained with Hoechst and CUG RNA foci were probed by Alexa555-$(CAG)_{10}$ fluorescent oligo. (A) Foci area per nuclear area and (B) number of nuclei per well (to assess ASO-associated toxicity) were quantified using Columbus Software. DMPK ASO was used as a positive control to measure assay quality and resulted in a Z' score of 0.84 using foci area per nuclear area in Supplementary Figure 3A, indicating an "excellent" separation between positive and negative controls.

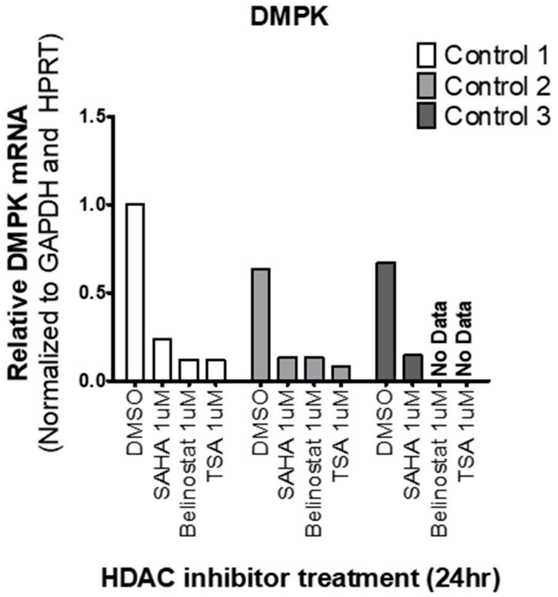

Fig. S4. Pan-HDAC inhibitors, vorinostat (SAHA), belinostat and TSA, all reduced DMPK mRNA levels in differentiated control myoblasts. Control myoblasts were serum-starved in 6-well plates for 7 days. Control cells were treated with DMSO alone 1 of vorinostat, belinostat and TSA. RNA was extracted (RNeasy micro kit, Qiagen) and reverse-transcribed to cDNA (iScript Advanced RT kit, BioRad). RT-qPCR was performed (iQ Sybr green supermix, BioRad) to assess mRNA levels of (A) *DMPK* and (B) *SERCA1* splicing. (n=1).

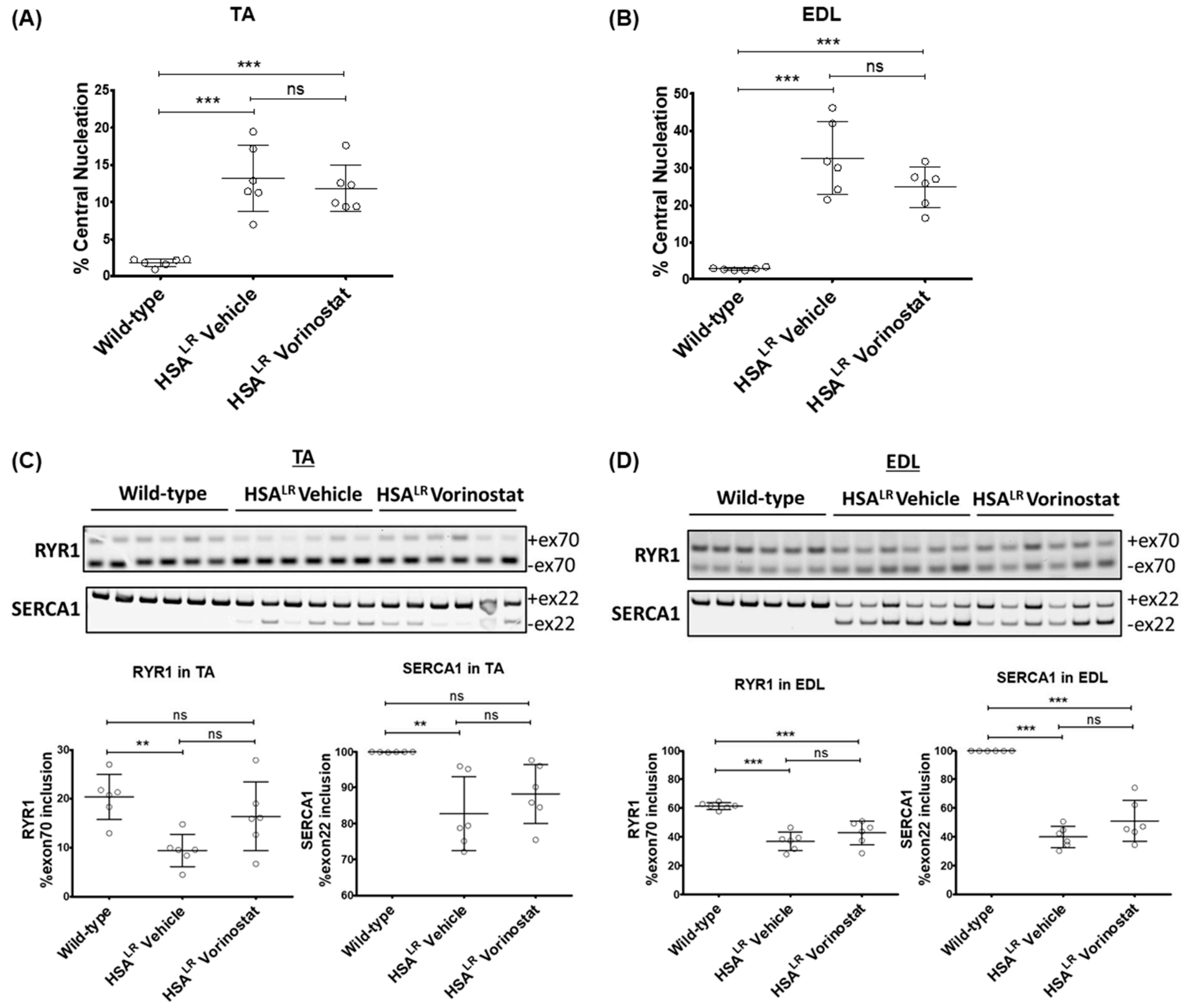

Fig. S5. Short-term treatment with low-dose (25 mg/kg) vorinostat showed a trend towards correcting disease pathology in HAS^{LR} mice. Mice at were injected (IP) daily with vehicle or 25 mg/kg vorinostat for 1 week. The mice were sacrifice by lethal injection and cervical dislocation. Skeletal muscle tissue from one hind leg was frozen in OCT for sectioning and imaging, and skeletal muscle tissue from the other hind leg was flash frozen in liquid nitrogen for RNA workup. (A-B) Analysis of central nucleation in TA and EDL muscle. Tissues flash frozen in OCT were sectioned at 10 µm thickness and subjected to H&E staining. Brightfield images were taken at 20x using EVOS cell imaging system and whole sections were manually counted for central nucleation. (C-D) Analysis of DM1 related spliceopathy in TA and EDL muscle. Flash frozen tissue was ground to a powder and a portion used for Trizol RNA extraction using the Purelink RNA mini kit (Invitrogen) and reverse transcribed to cDNA (iScript Advanced RT kit, BioRad). RT-sqPCR products using cDNA from (C) TA and (D) EDL were resolved on 7% acrylamide gel, stained with Gel Red and imaged using the ChemiDoc (BioRad). Quantification of transcript ratios was done using ImageLab (BioRad). (n=6, two-way ANOVA; error bars represent SD). *p< 0.05, **p< 0.01, ***p< 0.001).

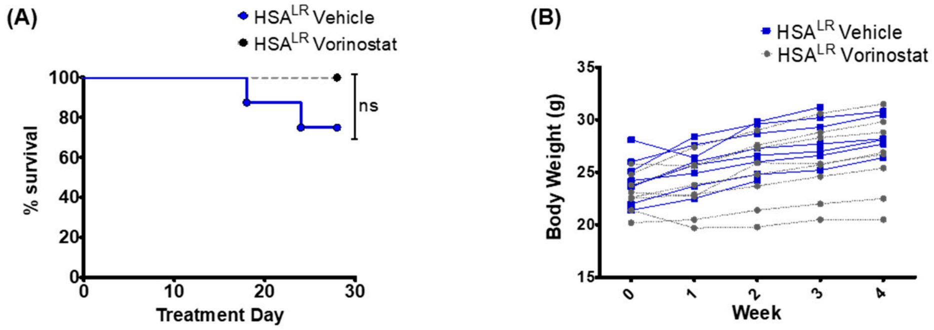

Fig. S6. Vorinostat is not toxic to DM1 HSA^{LR} mouse model. Mice at approximately 4 weeks of age were injected (IP) daily with vehicle or 50 mg/kg vorinostat for 4 weeks. The mice were sacrifice by lethal injection and cervical dislocation. Skeletal muscle tissue from one hind leg was frozen in OCT for sectioning and imaging, and skeletal muscle tissue from the other hind leg was flash frozen in liquid nitrogen for RNA workup. (A) Survival plot and (B) body weight of the mice was used to assess toxicity of the drug.

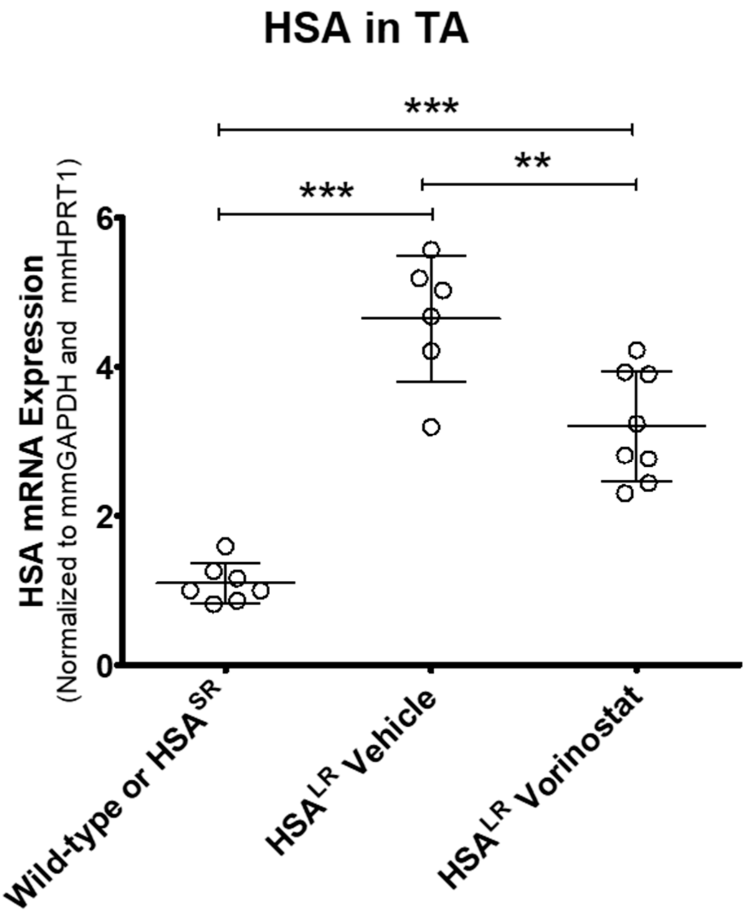

Fig. S7. Vorinostat reduced HSA mRNA levels in the TA muscle of DM1 HSALR mouse model. Mice at approximately 4 weeks of age were injected (IP) daily with vehicle or 50 mg/kg vorinostat for 4 weeks. The mice were sacrifice by lethal injection and cervical dislocation. Skeletal muscle tissue from one hind leg was frozen in OCT for sectioning and imaging, and skeletal muscle tissue from the other hind leg was flash frozen in liquid nitrogen for RNA workup. Flash frozen tissue was ground to a powder and a portion used for Trizol RNA extraction using the Purelink RNA mini kit (Invitrogen) and reverse transcribed to cDNA (iScript Advanced RT kit, BioRad). RT-qPCR was performed to quantity HSA mRNA levels in the TA muscle, relative to mouse GAPDH and HPRT (mmGAPDH and mmHPRT, respectively). (n ranges from 6 to 8, two-way ANOVA; error bars represent SD). $^{*}p < 0.05$, $^{**}p < 0.01$, $^{***}p < 0.001$.

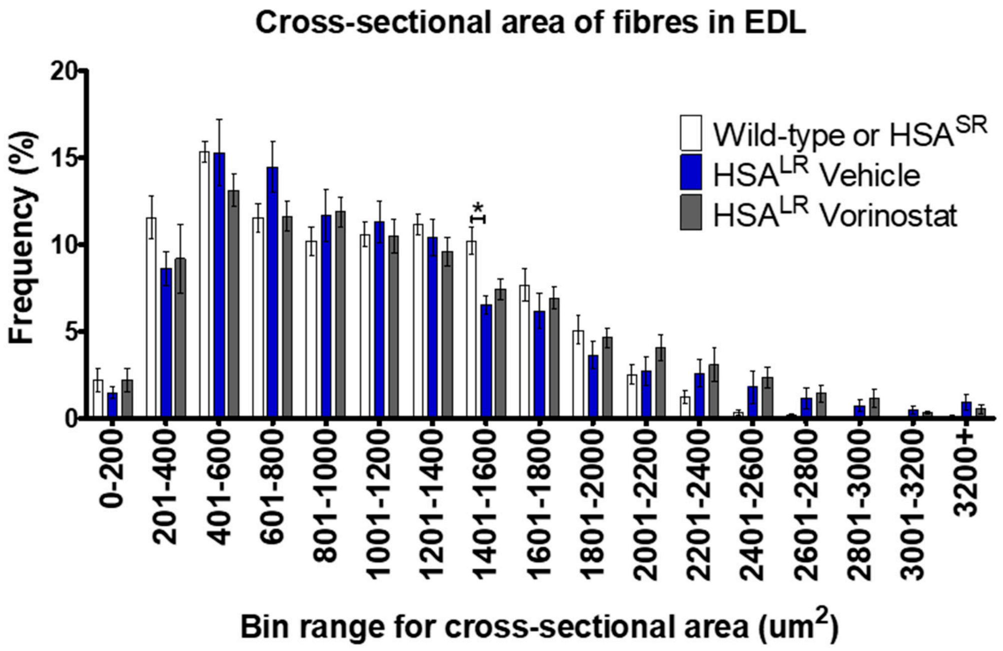

Fig. S8. Vorinostat does not correct trends of hypertrophy in EDL muscle of DM1 HSALR mouse model. Mice at approximately 4 weeks of age were injected (IP) daily with vehicle or 50 mg/kg vorinostat for 4 weeks. The mice were sacrifice by lethal injection and cervical dislocation. Skeletal muscle tissue from one hind leg was frozen in OCT for sectioning and imaging. Laminin-stained fibre images were used to quantify cross-sectional area (CSA); a combination of automated tracing using the open-CSAM plugin and manual tracing in Image J was used. In order to represent fibre-size distribution, the CSAs were binned every 200 μm^2 and % frequency of each bin was calculated relative to total number of lamini-positive fibres. (n 10 ranges from 6 to 8, two-way ANOVA; error bars represent SD). *$p< 0.05$, **$p< 0.01$, ***$p< 0.001$.

Table S1. Summary of FDA small-molecule screen in differentiated $DM1_{3600CTG}$ myoblasts. $DM1_{3600CTG}$ myoblasts were serum-starved in 384-well plates for 7 days and treated with 2 μM of drugs for 24 h. Post-treatment, cells were fixed with 4% PFA, DNA was stained with Hoechst and CUG RNA foci were probed by Alexa555-$(CAG)_{10}$. Foci area per nuclear area and number of nuclei per well were quantified using Columbus and normalized to DMSO control data per plate. Data is presented as the average fold-change relative to DMSO treatment (n=3). SCREEN-WELL® FDA approved drug library V2 catalog number BML-2843 (Enzo Life Sciences, Farmingdale, NY, USA). The rankings in Table S1 are based on the reduction in foci per nuclear area (green column).

Plate ID	Drug #	Drug Name	Well Name	Fold change based on DMSO mean (foci)		Fold change based on DMSO mean (nuclei)	
				Average	Stdev	Average	Stdev
cFDA800_1	141	Auranofin	G4	0.323	0.416	0.004	0.003
cFDA800_1	183	Vorinostat	K7	0.481	0.04	0.806	0.08
cFDA800_2	409	Arsenic Trioxide	C14	0.492	0.069	0.318	0.039
cFDA800_1	189	Gemcitabine·HCl	K19	0.618	0.038	0.87	0.019
cFDA800_1	170	Bortezomib	I21	0.673	0.023	0.729	0.045
cFDA800_2	437	Busulfan	F10	0.691	0.037	0.478	0.089
cFDA800_1	194	Clofarabine	L9	0.726	0.012	0.933	0.018
cFDA800_1	256	Disulfiram	J14	0.73	0.099	0.552	0.168
cFDA800_2	368	Misoprostol	E17	0.749	0.053	0.986	0.03
cFDA800_1	156	Mitomycin C	H14	0.755	0.015	0.922	0.096
cFDA800_1	248	Cytarabine	I18	0.763	0.041	0.911	0.032
cFDA800_1	305	Mitoxantrone·HCl	O12	0.764	0.218	0.445	0.019
cFDA800_3	703	Podofilox	H21	0.766	0.047	0.96	0.121
cFDA800_2	410	Artemether	C16	0.774	0.038	0.99	0.024
cFDA800_2	423	Benazepril·HCl	D22	0.779	0.037	1.101	0.021
cFDA800_1	244	Clobetasol Propionate	I10	0.788	0.033	1.07	0.035
cFDA800_3	322	Penicillin V Potassium	M5	0.8	0.42	1.007	0.048
cFDA800_2	370	Cilastatin·Na	E21	0.803	0.045	1.004	0.033
cFDA800_2	453	Cefprozil	G22	0.806	0.045	0.928	0.091
cFDA800_2	445	Cefadroxil	G6	0.81	0.016	0.921	0.058
cFDA800_2	492	Dantrolene·Na	K19	0.812	0.018	1.054	0.057
cFDA800_2	595	Ketorolac Tromethamine	N6	0.816	0.05	0.961	0.02
cFDA800_3	789	Ursodiol	I13	0.816	0.204	1.047	0.107
cFDA800_2	422	Beclomethasone Dipropionate	D20	0.827	0.077	0.965	0.112
cFDA800_3	657	Mometasone Furoate	D9	0.827	0.095	0.982	0.104
cFDA800_2	525	Droperidol	O5	0.83	0.08	1.013	0.023
cFDA800_2	383	Cyproheptadine·HCl Sesquihydrate	G7	0.833	0.034	1.121	0.122
cFDA800_3	779	Tretinoin	H14	0.835	0.055	1.114	0.249
cFDA800_3	311	Norfloxacin	L4	0.843	0.189	1.138	0.192

cFDA800_3	780	Triamcinolone Acetonide	H16	0.844	0.016	0.975	0.11
cFDA800_1	107	Dinoprostone	C16	0.849	0.041	0.948	0.076
cFDA800_1	209	Vincristine Sulfate	M19	0.849	0.072	0.858	0.009
cFDA800_3	81	Nisoldipine	K4	0.851	0.241	1.048	0.086
cFDA800_1	135	Ethacrynic Acid	F12	0.851	0.02	0.937	0.06
cFDA800_1	133	Dexamethasone	F8	0.853	0.059	1.069	0.02
cFDA800_1	120	Gabapentin	D22	0.856	0.223	0.97	0.054
cFDA800_3	1	Clindamycin·HCl	K3	0.858	0.231	1.063	0.188
cFDA800_1	300	Methylprednisolone	N22	0.864	0.038	1.024	0.084
cFDA800_1	273	Fluocinolone Acetonide	L8	0.865	0.042	0.991	0.05
cFDA800_2	577	Homatropine Methylbromide	L10	0.869	0.016	0.968	0.05
cFDA800_2	381	Capecitabine	G3	0.877	0.048	1.079	0.117
cFDA800_1	199	Adapalene	L19	0.88	0.035	0.868	0.036
cFDA800_2	332	Ramipril	B5	0.88	0.041	1.189	0.046
cFDA800_1	167	Calcipotriene	I15	0.882	0.026	1.02	0.099
cFDA800_1	227	Betamethasone	O15	0.883	0.049	0.995	0.088
cFDA800_2	341	Sulfasalazine	C3	0.884	0.04	0.978	0.065
cFDA800_2	459	Cephalexin Monohydrate	H14	0.885	0.061	0.891	0.049
cFDA800_2	505	Desoximetasone	M5	0.89	0.051	1.014	0.019
cFDA800_1	283	Hydrocortisone Acetate	M8	0.895	0.067	1.051	0.099
cFDA800_1	53	(±) Isoproterenol·HCl	F7	0.896	0.029	0.852	0.016
cFDA800_3	614	Mafenide·HCl	N4	0.899	0.336	1.119	0.137
cFDA800_3	6	Mycophenolic Acid	K13	0.901	0.069	1.054	0.218
cFDA800_1	63	Epinephrine (L-(-)-Epinephrine-(+)-Bitartrate)	G7	0.902	0.019	0.856	0.073
cFDA800_1	32	Salbutamol Hemisulfate	D5	0.903	0.016	0.86	0.03
cFDA800_3	7	Sirolimus (Rapamycin)	K15	0.904	0.091	1.134	0.227
cFDA800_3	698	Phenylephrine	H11	0.907	0.082	0.632	0.51
cFDA800_1	282	Hydrocortisone	M6	0.912	0.066	1.007	0.198
cFDA800_2	378	Miglustat (N-Butyldeoxynojirimycin·HCl)	F17	0.916	0.096	0.881	0.057
cFDA800_3	740	Sitagliptin Phosphate	D16	0.916	0.058	1.089	0.13
cFDA800_3	783	Trientine Dihydrochloride	H22	0.917	0.061	1.101	0.179
cFDA800_3	321	Pentoxifylline	M3	0.92	0.23	0.722	0.499
cFDA800_1	97	Bleomycin Sulfate	B16	0.921	0.06	0.873	0.076
cFDA800_1	193	Mycophenolate Mofetil	L7	0.921	0.021	0.917	0.055
cFDA800_1	34	Dobutamine·HCl	D9	0.923	0.013	0.997	0.061
cFDA800_1	77	Docetaxel (Taxotere)	H15	0.926	0.021	0.813	0.017
cFDA800_3	671	Nilutamide	E17	0.926	0.108	0.966	0.056
cFDA800_1	59	Risperidone	F19	0.927	0.06	0.965	0.065
cFDA800_3	672	Nitazoxanide	E19	0.927	0.006	0.942	0.082
cFDA800_3	758	Terbutaline Hemisulfate	F12	0.927	0.031	0.839	0.074

cFDA800_2	352	Amoxicillin	D5	0.928	0.077	0.918	0.069
cFDA800_3	680	Oxazepam	F15	0.928	0.06	0.889	0.234
cFDA800_3	692	Pentamidine Isethionate	G19	0.928	0.063	0.975	0.02
cFDA800_3	725	Repaglinide	C6	0.928	0.064	0.988	0.165
cFDA800_2	405	Aminohippurate·Na	C6	0.93	0.066	0.913	0.071
cFDA800_3	753	Telithromycin	E22	0.93	0.071	0.992	0.236
cFDA800_3	729	Rifaximin	C14	0.931	0.17	0.895	0.086
cFDA800_2	407	Amlexanox	C10	0.932	0.068	0.965	0.134
cFDA800_3	535	Eplerenone	N5	0.932	0.238	1.085	0.149
cFDA800_2	357	Venlafaxine·HCl	D15	0.933	0.08	0.863	0.121
cFDA800_1	207	Vinorelbine	M15	0.934	0.073	0.88	0.033
cFDA800_1	208	Salmeterol	M17	0.935	0.064	0.89	0.081
cFDA800_2	350	Toremifene Base	C21	0.936	0.046	0.962	0.07
cFDA800_1	110	Dofetilide	C22	0.937	0.079	1.064	0.059
cFDA800_1	175	Bexarotene	J11	0.938	0.046	0.932	0.126
cFDA800_2	359	Ketotifen Fumarate	D19	0.938	0.026	0.856	0.074
cFDA800_1	129	Calcitriol	E20	0.94	0.011	0.92	0.027
cFDA800_3	662	Naratriptan·HCl	D19	0.941	0.057	1.08	0.199
cFDA800_3	727	Rifabutin	C10	0.941	0.09	1.046	0.185
cFDA800_3	763	Theophylline	F22	0.942	0.065	1.032	0.125
cFDA800_3	773	Tolvaptan	G22	0.942	0.032	1.078	0.128
cFDA800_3	696	Perphenazine	H7	0.943	0.085	1.029	0.149
cFDA800_3	324	Prednisolone	M9	0.945	0.075	1.101	0.253
cFDA800_2	435	Budesonide	F6	0.945	0.052	0.948	0.026
cFDA800_3	694	Perindopril Erbumine	H3	0.945	0.054	1.027	0.051
cFDA800_1	228	Bisacodyl	O17	0.947	0.082	0.728	0.057
cFDA800_3	688	Pemetrexed Disodium	G11	0.947	0.088	1.073	0.096
cFDA800_3	778	Trazodone·HCl	H12	0.947	0.084	0.962	0.104
cFDA800_3	782	Triazolam	H20	0.947	0.057	1.035	0.153
cFDA800_3	748	Sunitinib Malate	E12	0.948	0.05	0.925	0.156
cFDA800_2	398	Almotriptan	B12	0.949	0.045	1.069	0.049
cFDA800_3	686	Paromomycin Sulfate	G7	0.95	0.086	0.937	0.017
cFDA800_3	746	Sulfamethoxazole	E8	0.95	0.051	0.958	0.098
cFDA800_1	106	Exemestane	C14	0.951	0.058	0.988	0.052
cFDA800_3	384	Abacavir Sulfate	M4	0.951	0.191	1.091	0.175
cFDA800_2	399	Alosetron·HCl	B14	0.951	0.041	1.003	0.061
cFDA800_3	754	Temazepam	F4	0.951	0.03	1.033	0.182
cFDA800_1	36	Maprotiline·HCl	D13	0.952	0.034	0.94	0.098
cFDA800_2	421	Balsalazide	D18	0.952	0.025	0.905	0.022
cFDA800_1	104	Idarubicin·HCl	C10	0.953	0.05	0.807	0.04

cFDA800_2	348	Tobramycin	C17	0.953	0.04	0.929	0.023
cFDA800_2	400	Blank	B16	0.953	0.011	1.041	0.015
cFDA800_3	625	Mequinol	O5	0.953	0.162	1.038	0.157
cFDA800_1	165	Sulindac	I11	0.954	0.045	0.996	0.073
cFDA800_1	128	Acitretin	E18	0.955	0.036	0.949	0.022
cFDA800_1	246	Crotamiton	I14	0.955	0.055	1.024	0.066
cFDA800_2	420	Baclofen	D16	0.955	0.048	0.889	0.096
cFDA800_3	743	Streptozocin	D22	0.955	0.066	1.075	0.153
cFDA800_3	790	Valganciclovir·HCl	I15	0.955	0.049	0.983	0.019
cFDA800_3	85	Azathioprine	K12	0.956	0.057	0.961	0.093
cFDA800_2	444	Cefaclor	G4	0.956	0.021	0.949	0.021
cFDA800_1	168	Zafirlukast	I17	0.957	0.02	0.999	0.089
cFDA800_2	443	Carmustine	F22	0.957	0.042	0.973	0.012
cFDA800_1	151	Simvastatin	H4	0.958	0.007	0.884	0.105
cFDA800_1	171	Diazoxide	J3	0.958	0.038	1.18	0.306
cFDA800_1	245	Orphenadrine Citrate	I12	0.958	0.026	0.957	0.017
cFDA800_1	249	Dacarbazine	I20	0.96	0.018	0.961	0.013
cFDA800_3	682	Oxybutynin Chloride	F19	0.96	0.106	0.812	0.233
cFDA800_1	39	Tropicamide	D19	0.961	0.032	0.896	0.075
cFDA800_2	356	Vecuronium Bromide	D13	0.961	0.044	0.907	0.054
cFDA800_3	635	Methocarbamol	B5	0.961	0.109	0.906	0.221
cFDA800_3	767	Tiagabine·HCl	G10	0.961	0.053	1.063	0.141
cFDA800_1	99	Tizanidine·HCl	B20	0.962	0.035	0.989	0.02
cFDA800_2	336	Propranolol·HCl	B13	0.962	0.063	0.979	0.056
cFDA800_2	396	Blank	B8	0.962	0.008	1.047	0.079
cFDA800_2	429	Bimatoprost	E14	0.962	0.054	0.928	0.071
cFDA800_3	681	Oxtriphylline	F17	0.962	0.024	1.021	0.232
cFDA800_3	690	Penicillamine (D-Penicillamine)	G15	0.962	0.091	0.972	0.091
cFDA800_2	413	Asenapine Maleate	C22	0.963	0.017	0.948	0.043
cFDA800_3	683	Oxytetracycline·HCl	F21	0.963	0.027	0.96	0.128
cFDA800_1	67	Amoxapine	G15	0.964	0.018	0.98	0.069
cFDA800_1	74	Clemastine Fumarate	H9	0.964	0.045	0.948	0.04
cFDA800_2	345	Tetracycline	C11	0.964	0.034	0.908	0.032
cFDA800_2	397	Alitretinoin	B10	0.964	0.035	1.054	0.052
cFDA800_3	742	Stavudine	D20	0.964	0.063	0.657	0.505
cFDA800_3	800	Ziprasidone	J15	0.964	0.061	1.118	0.154
cFDA800_1	60	Diphenhydramine·HCl	F21	0.965	0.032	0.929	0.096
cFDA800_1	177	Celecoxib	J15	0.965	0.073	1.001	0.115
cFDA800_2	340	Sulfadiazine	B21	0.965	0.035	1.011	0.021
cFDA800_2	369	Argatroban	E19	0.965	0.037	0.961	0.085

cFDA800_3	689	Pemirolast Potassium	G13	0.965	0.033	1.016	0.132
cFDA800_3	724	Regadenoson	C4	0.965	0.05	1.065	0.152
cFDA800_2	365	Amrinone	E11	0.966	0.063	0.977	0.084
cFDA800_3	673	Nitisinone	E21	0.966	0.08	1.059	0.133
cFDA800_3	702	Blank	H19	0.966	0.095	0.986	0.155
cFDA800_3	749	Tacrolimus (Fk506)	E14	0.966	0.077	1.007	0.211
cFDA800_1	178	Levetiracetam	J17	0.967	0.017	0.968	0.07
cFDA800_2	457	Cefuroxime Axetil	H10	0.967	0.036	0.971	0.041
cFDA800_2	576	Hexachlorophene	L8	0.967	0.041	0.949	0.063
cFDA800_3	659	Nadolol	D13	0.967	0.101	0.625	0.496
cFDA800_1	109	Anagrelide	C20	0.968	0.031	0.919	0.03
cFDA800_2	331	Ranolazine·2HCl	B3	0.968	0.028	1.039	0.042
cFDA800_2	339	Streptomycin Sulfate	B19	0.968	0.028	1.071	0.045
cFDA800_3	676	Nortriptyline·HCl	F7	0.968	0.069	0.982	0.201
cFDA800_3	687	Pazopanib·HCl	G9	0.968	0.051	0.616	0.527
cFDA800_1	41	Ivermectin	E3	0.969	0.008	1.008	0.028
cFDA800_2	454	Ceftibuten	H4	0.969	0.044	1.019	0.033
cFDA800_3	674	Nitrofurantoin	F3	0.969	0.072	1.035	0.251
cFDA800_3	741	Sorafenib Tosylate	D18	0.969	0.077	1.037	0.123
cFDA800_3	744	Sulconazole Nitrate	E4	0.969	0.133	0.615	0.498
cFDA800_1	79	Tolcapone	H19	0.97	0.045	0.997	0.084
cFDA800_2	408	Amphotericin B	C12	0.97	0.051	0.913	0.086
cFDA800_3	734	Rosuvastatin Calcium	D4	0.97	0.028	1.004	0.161
cFDA800_1	139	Bumetanide	F20	0.971	0.032	0.977	0.144
cFDA800_3	760	Testosterone Enanthate	F16	0.971	0.048	1.024	0.136
cFDA800_1	16	Quinine·HCl·H2O	B13	0.972	0.02	1.052	0.136
cFDA800_2	463	Chlorothiazide	H22	0.972	0.016	0.974	0.07
cFDA800_3	663	Natamycin	D21	0.972	0.052	0.914	0.15
cFDA800_3	768	Tigecycline	G12	0.972	0.003	1.024	0.099
cFDA800_3	774	Topiramate	H4	0.972	0.05	1.021	0.036
cFDA800_1	35	Sotalol·HCl	D11	0.973	0.028	0.939	0.022
cFDA800_1	154	Rifampin (Rifampicin)	H10	0.973	0.025	0.939	0.066
cFDA800_3	231	Carbamazepine	L3	0.973	0.026	0.796	0.403
cFDA800_1	241	Clarithromycin	I4	0.973	0.034	1.133	0.049
cFDA800_3	320	Penciclovir	L22	0.973	0.033	1.171	0.349
cFDA800_2	337	Scopolamine·HBr	B15	0.973	0.037	0.957	0.043
cFDA800_2	411	Articaine·HCl	C18	0.973	0.065	0.983	0.032
cFDA800_3	653	Mirtazapine	C21	0.973	0.078	1.005	0.113
cFDA800_3	655	Modafinil (Schedule Iv)	D5	0.973	0.037	1.051	0.129
cFDA800_3	695	Permethrin	H5	0.973	0.05	0.755	0.408

cFDA800_1	111	Erlotinib	D4	0.974	0.053	0.993	0.052
cFDA800_3	645	Blank	C5	0.974	0.071	0.812	0.355
cFDA800_3	776	Trandolapril	H8	0.974	0.015	1.052	0.194
cFDA800_1	52	Nateglinide	F5	0.975	0.03	0.911	0.012
cFDA800_2	353	Tramadol·HCl	D7	0.975	0.031	0.856	0.058
cFDA800_2	441	Carglumic Acid	F18	0.975	0.043	0.921	0.087
cFDA800_2	581	Hydroxocobalamin·HCl	L18	0.975	0.064	0.993	0.118
cFDA800_3	643	Metolazone	B21	0.975	0.072	1.159	0.153
cFDA800_3	771	Tirofiban·HCl	G18	0.975	0.073	0.671	0.57
cFDA800_2	354	Trimethoprim	D9	0.976	0.028	0.85	0.038
cFDA800_2	402	Amcinonide	B20	0.976	0.031	1.038	0.053
cFDA800_3	675	Nizatidine	F5	0.976	0.118	0.961	0.068
cFDA800_3	786	Trimethobenzamide·HCl	I7	0.976	0.043	0.998	0.052
cFDA800_3	797	Warfarin·Na	J9	0.976	0.035	0.966	0.173
cFDA800_1	25	Clonidine·HCl	C11	0.977	0.046	0.947	0.022
cFDA800_1	143	Tranylcypromine Hemisulfate	G8	0.977	0.046	0.939	0.08
cFDA800_2	333	Ribavirin	B7	0.977	0.011	1.15	0.228
cFDA800_2	461	Blank	H18	0.977	0.035	1.059	0.093
cFDA800_3	684	Paliperidone	G3	0.977	0.042	1.118	0.175
cFDA800_3	787	Trimipramine Maleate	I9	0.977	0.055	1.069	0.251
cFDA800_3	796	Voriconazole	J7	0.977	0.013	1.048	0.08
cFDA800_1	142	Captopril	G6	0.978	0.042	1.055	0.079
cFDA800_2	403	Amikacin Disulfate	B22	0.978	0.063	1.037	0.04
cFDA800_2	412	L-Ascorbic Acid	C20	0.978	0.044	0.961	0.104
cFDA800_2	530	Econazole Nitrate	O15	0.978	0.044	1.026	0.083
cFDA800_3	728	Rifapentine	C12	0.978	0.049	1	0.119
cFDA800_1	18	Phenytoin	B17	0.979	0.032	1.135	0.162
cFDA800_1	150	Sodium Phenylbutyrate	G22	0.979	0.012	0.963	0.093
cFDA800_1	153	Raloxifene·HCl	H8	0.979	0.024	1.051	0.088
cFDA800_2	338	Spironolactone	B17	0.979	0.07	1.064	0.037
cFDA800_2	375	Aprepitant	F11	0.979	0.041	1.044	0.201
cFDA800_3	387	Acebutolol·HCl	M10	0.979	0.05	0.986	0.076
cFDA800_2	566	Formoterol	K8	0.979	0.062	0.953	0.111
cFDA800_3	665	Nelarabine	E5	0.979	0.04	1.036	0.161
cFDA800_3	732	Ropinirole·HCl	C20	0.979	0.033	1.063	0.166
cFDA800_1	28	Emtricitabine	C17	0.98	0.04	0.87	0.053
cFDA800_1	31	(S)-Timolol Maleate	D3	0.98	0.017	1.034	0.104
cFDA800_1	91	Candesartan	B4	0.98	0.036	1.122	0.102
cFDA800_2	344	Terazosin·HCl	C9	0.98	0.036	1.029	0.005
cFDA800_2	419	Bacitracin	D14	0.98	0.049	0.909	0.075

cFDA800_2	481	Cloxacillin·Na	J17	0.98	0.005	0.977	0.024
cFDA800_3	651	Miglitol	C17	0.98	0.053	1.017	0.15
cFDA800_3	699	Phytonadione	H13	0.98	0.03	0.965	0.129
cFDA800_3	770	Tiopronin	G16	0.98	0.044	0.973	0.07
cFDA800_1	61	Promethazine·HCl	G3	0.981	0.048	0.968	0.055
cFDA800_1	118	Nimodipine	D18	0.981	0.05	0.935	0.057
cFDA800_1	260	Esomeprazole Potassium	J22	0.981	0.044	0.956	0.091
cFDA800_2	401	Ambrisentan	B18	0.981	0.032	1.002	0.093
cFDA800_1	19	Procainamide·HCl	B19	0.982	0.013	1.108	0.139
cFDA800_1	26	Guanabenz Acetate	C13	0.982	0.026	0.976	0.047
cFDA800_1	27	Dihydroergotamine Mesylate	C15	0.982	0.086	1.001	0.044
cFDA800_3	88	Sertaconazole	K18	0.982	0.165	1.072	0.043
cFDA800_1	131	Cromolyn·Na (Disodium Cromoglycate)	F4	0.982	0.046	0.989	0.137
cFDA800_3	656	Moexipril·HCl	D7	0.982	0.052	1.125	0.258
cFDA800_3	677	Olsalazine·Na	F9	0.982	0.064	1.007	0.086
cFDA800_3	693	Pentostatin	G21	0.982	0.017	0.977	0.053
cFDA800_3	720	Rabeprazole·Na	B16	0.982	0.032	0.972	0.22
cFDA800_1	68	Metoclopramide·HCl	G17	0.983	0.036	1.005	0.054
cFDA800_3	86	Sildenafil Citrate	K14	0.983	0.067	0.97	0.076
cFDA800_1	124	Latanoprost	E10	0.983	0.045	1.066	0.027
cFDA800_2	346	Temozolomide	C13	0.983	0.046	0.971	0.045
cFDA800_2	395	Adenosine	B6	0.983	0.022	1.192	0.049
cFDA800_2	438	Butorphanol-(+)-Tartrate (Schedule Iv)	F12	0.983	0.081	0.936	0.073
cFDA800_2	490	Dactinomycin (= Actinomycin D)	K15	0.983	0.054	0.962	0.116
cFDA800_3	650	Midodrine·HCl	C15	0.983	0.056	1.062	0.119
cFDA800_3	679	Oxaprozin	F13	0.983	0.055	0.933	0.133
cFDA800_3	715	Protriptyline·HCl	B6	0.983	0.04	0.932	0.113
cFDA800_3	718	Pyrimethamine	B12	0.983	0.064	1.036	0.078
cFDA800_1	13	Tolbutamide	B7	0.984	0.029	1.052	0.14
cFDA800_1	44	Zonisamide	E9	0.984	0.034	1.056	0.08
cFDA800_2	360	Naloxone·HCl	D21	0.984	0.017	0.862	0.093
cFDA800_3	745	Sulfacetamide·Na	E6	0.984	0.073	1.018	0.143
cFDA800_3	765	Thiotepa	G6	0.984	0.078	1.056	0.138
cFDA800_2	428	Bethanechol Chloride	E12	0.985	0.078	0.947	0.086
cFDA800_2	440	Carbinoxamine Maleate	F16	0.985	0.007	0.893	0.056
cFDA800_2	473	Cisplatin (Cis-Diamineplatinum(Ii) Dichloride)	I21	0.985	0.072	1.003	0.133
cFDA800_3	794	Varenicline Tartrate	J3	0.985	0.019	1.108	0.055
cFDA800_1	47	Zolmitriptan	E15	0.986	0.013	0.935	0.103
cFDA800_1	64	Norepinephrine Bitartrate Monohydrate	G9	0.986	0.016	0.962	0.024
cFDA800_1	160	Cetirizine 2HCl	H22	0.986	0.031	0.922	0.109

cFDA800_2	448	Cefditoren Pivoxil	G12	0.986	0.037	0.945	0.03
cFDA800_2	511	Diatrizoate Meglumine	M17	0.986	0.017	0.96	0.031
cFDA800_3	615	Malathion	N6	0.986	0.072	1.005	0.153
cFDA800_3	660	Nafcillin·Na	D15	0.986	0.067	1.054	0.204
cFDA800_3	751	Tazarotene	E18	0.986	0.061	1.034	0.142
cFDA800_1	17	Propafenone·HCl	B15	0.987	0.028	0.964	0.005
cFDA800_1	22	Rosiglitazone	C5	0.987	0.097	1.039	0.077
cFDA800_1	116	Diltiazem·HCl	D14	0.987	0.023	0.944	0.043
cFDA800_1	155	Etoposide	H12	0.987	0.072	0.918	0.025
cFDA800_3	233	Ceftazidime	L7	0.987	0.055	0.924	0.134
cFDA800_3	733	Ropivacaine·HCl Monohydrate	C22	0.987	0.057	1.023	0.149
cFDA800_1	71	Famotidine	H3	0.988	0.022	0.996	0.077
cFDA800_2	418	Azelastine·HCl	D12	0.988	0.027	0.928	0.034
cFDA800_2	442	Blank	F20	0.988	0.022	0.882	0.081
cFDA800_1	21	Flecainide Acetate	C3	0.989	0.027	1.005	0.011
cFDA800_1	80	Olmesartan	H21	0.989	0.064	0.891	0.095
cFDA800_1	176	Tranexamic Acid	J13	0.989	0.032	0.971	0.067
cFDA800_1	218	Amifostine	N17	0.989	0.039	0.926	0.085
cFDA800_1	242	Clomiphene Citrate	I6	0.989	0.061	1.016	0.062
cFDA800_1	243	Clopidogrel Hydrogen Sulfate	I8	0.989	0.028	0.986	0.08
cFDA800_1	267	Fenofibrate	K16	0.989	0.019	0.984	0.091
cFDA800_2	342	Tamsulosin·HCl	C5	0.989	0.089	0.957	0.022
cFDA800_2	363	Tiotropium Bromide Monohydrate	E7	0.989	0.045	0.979	0.11
cFDA800_2	371	Butoconazole Nitrate	F3	0.989	0.029	0.943	0.047
cFDA800_2	434	Brompheniramine Maleate	F4	0.989	0.05	0.922	0.101
cFDA800_2	458	Cefuroxime·Na	H12	0.989	0.066	0.969	0.103
cFDA800_2	571	Glycopyrrolate Iodide	K18	0.989	0.013	0.999	0.024
cFDA800_3	691	Penicillin G Potassium (Benzylpenicillin)	G17	0.989	0.103	1.026	0.061
cFDA800_3	739	Silver Sulfadiazine	D14	0.989	0.08	1.084	0.219
cFDA800_3	9	Amiodarone·HCl	K19	0.99	0.047	0.865	0.142
cFDA800_1	11	Pimozide	B3	0.99	0.038	1.1	0.001
cFDA800_1	40	Pancuronium·2Br	D21	0.99	0.04	0.944	0.074
cFDA800_2	404	Aminocaproic Acid	C4	0.99	0.07	1.012	0.122
cFDA800_2	486	Cyclobenzaprine·HCl	K7	0.99	0.08	1.058	0.07
cFDA800_3	634	Methenamine Hippurate	B3	0.99	0.05	1.168	0.142
cFDA800_1	37	Pilocarpine·HCl	D15	0.991	0.005	0.976	0.089
cFDA800_1	45	Zoledronic Acid Monohydrate	E11	0.991	0.039	0.973	0.017
cFDA800_1	69	Nalbuphine·HCl Dihydrate	G19	0.991	0.055	0.951	0.049
cFDA800_1	127	Clozapine	E16	0.991	0.022	0.956	0.016
cFDA800_1	250	Danazol	I22	0.991	0.014	1.007	0.037

cFDA800_2	415	Atorvastatin Calcium	D6	0.991	0.007	0.967	0.041
cFDA800_1	98	Guanfacine·HCl	B18	0.992	0.032	0.985	0.04
cFDA800_1	101	Flumazenil	C4	0.992	0.056	1.012	0.079
cFDA800_1	112	Tacrine·HCl	D6	0.992	0.021	0.926	0.129
cFDA800_1	184	Didanosine	K9	0.992	0.028	0.961	0.024
cFDA800_1	251	Desloratadine	J4	0.992	0.064	1.071	0.119
cFDA800_1	261	Estradiol	K4	0.992	0.043	0.991	0.059
cFDA800_2	351	Tolmetin·Na	D3	0.992	0.035	0.945	0.057
cFDA800_2	501	Demeclocycline·HCl	L17	0.992	0.059	0.945	0.067
cFDA800_2	559	Fluocinonide	J14	0.992	0.065	0.931	0.08
cFDA800_3	738	Sertraline·HCl	D12	0.992	0.056	1.012	0.176
cFDA800_3	755	Temsirolimus	F6	0.992	0.077	0.906	0.198
cFDA800_3	791	Valproate·Na	I17	0.992	0.058	1.079	0.139
cFDA800_3	792	Valsartan	I19	0.992	0.051	0.689	0.552
cFDA800_3	5	Lincomycin·HCl	K11	0.993	0.036	1.007	0.108
cFDA800_1	117	Nifedipine	D16	0.993	0.034	0.978	0.052
cFDA800_2	366	Milrinone	E13	0.993	0.035	1.041	0.078
cFDA800_2	406	Aminolevulinic Acid·HCl	C8	0.993	0.098	0.988	0.102
cFDA800_2	472	Cisatracurium Besylate	I19	0.993	0.017	0.996	0.059
cFDA800_3	764	Thioguanine (6-Thioguanine)	G4	0.993	0.053	1.056	0.12
cFDA800_1	20	Lidocaine·HCl·H2O	B21	0.994	0.042	0.941	0.068
cFDA800_1	48	Memantine·HCl	E17	0.994	0.019	0.977	0.087
cFDA800_3	83	Lovastatin	K8	0.994	0.038	1.027	0.042
cFDA800_1	114	Amiloride·HCl·2H2O	D10	0.994	0.012	0.997	0.083
cFDA800_1	181	Bicalutamide	K3	0.994	0.019	1.069	0.12
cFDA800_2	349	Topotecan·HCl	C19	0.994	0.016	0.959	0.031
cFDA800_2	367	Alprostadil	E15	0.994	0.017	0.946	0.063
cFDA800_3	540	Eszopiclone	N15	0.994	0.062	1.048	0.179
cFDA800_3	769	Tiludronate Disodium	G14	0.994	0.081	1.099	0.142
cFDA800_3	784	Trihexyphenidyl·HCl	I3	0.994	0	0.959	0.142
cFDA800_1	14	Glipizide	B9	0.995	0.025	0.992	0.12
cFDA800_1	102	Gefitinib	C6	0.995	0.05	0.872	0.048
cFDA800_2	455	Ceftizoxim·Na	H6	0.995	0.037	1.056	0.073
cFDA800_3	637	Methoxsalen (Xanthotoxin)	B9	0.995	0.075	1.079	0.154
cFDA800_3	639	Methsuximide	B13	0.995	0.084	1.044	0.129
cFDA800_1	95	Eprosartan Mesylate	B12	0.996	0.054	1.028	0.113
cFDA800_1	169	Zileuton	I19	0.996	0.058	0.944	0.074
cFDA800_1	268	Finasteride	K18	0.996	0.053	0.932	0.048
cFDA800_2	483	Colistimethate·Na	J21	0.996	0.013	1.013	0.068
cFDA800_2	545	Ethosuximide	I6	0.996	0.036	1.026	0.1

cFDA800_1	258	Doxycycline Monohydrate	J18	0.997	0.015	0.909	0.058
cFDA800_2	347	Tinidazole	C15	0.997	0.008	0.958	0.028
cFDA800_3	640	Methyclothiazide	B15	0.997	0.045	1.118	0.114
cFDA800_3	642	Methylergonovine Maleate	B19	0.997	0.057	1.117	0.157
cFDA800_3	775	Torsemide	H6	0.997	0.063	1.052	0.045
cFDA800_1	66	Imipramine·HCl	G13	0.998	0.095	1.005	0.041
cFDA800_1	76	Linezolid	H13	0.998	0.058	1.033	0.12
cFDA800_1	108	Metformin·HCl	C18	0.998	0.078	0.925	0.062
cFDA800_1	125	Alfuzosin	E12	0.998	0.051	0.95	0.032
cFDA800_1	137	Naproxen	F16	0.998	0.058	0.91	0.056
cFDA800_3	319	Paroxetine·HCl	L20	0.998	0.093	1.016	0.151
cFDA800_2	425	Bendroflumethiazide	E6	0.998	0.035	0.956	0.141
cFDA800_2	469	Cidofovir	I13	0.998	0.063	0.979	0.062
cFDA800_2	475	Clavulanate Potassium	J5	0.998	0.024	1.143	0.235
cFDA800_3	646	Mexiletine·HCl	C7	0.998	0.051	1.085	0.205
cFDA800_3	84	Lamotrigine	K10	0.999	0.036	1.016	0.212
cFDA800_1	119	Verapamil·HCl	D20	0.999	0.043	0.925	0.06
cFDA800_1	123	Trifluoperazine·HCl	E8	0.999	0.047	0.944	0.035
cFDA800_1	130	Ketoconazole	E22	0.999	0.051	0.926	0.035
cFDA800_1	145	Moxifloxacin·HCl	G12	0.999	0.046	0.961	0.096
cFDA800_1	147	Ketoprofen	G16	0.999	0.016	1.017	0.06
cFDA800_3	385	Acamprosate	M6	0.999	0.034	1.036	0.241
cFDA800_3	700	Pimecrolimus	H15	0.999	0.024	1.054	0.115
cFDA800_3	716	Pyrazinamide	B8	0.999	0.093	1.104	0.142
cFDA800_3	766	Blank	G8	0.999	0.071	1.03	0.231
cFDA800_3	772	Tolterodine Tartrate	G20	0.999	0.046	0.974	0.149
cFDA800_3	3	Cyclosporine A	K7	1	0.039	1.06	0.124
cFDA800_1	140	Neomycin Sulfate	F22	1	0.058	0.951	0.088
cFDA800_2	462	Chlorhexidine Dihydrochloride	H20	1	0.051	1.009	0.068
cFDA800_3	757	Tenofovir	F10	1	0.061	1.039	0.103
cFDA800_3	761	Tetrabenazine	F18	1	0.042	1.071	0.126
cFDA800_1	30	Caffeine	C21	1.001	0.007	1	0.051
cFDA800_1	96	Entacapone	B14	1.001	0.056	1.101	0.008
cFDA800_3	234	Chloramphenicol	L9	1.001	0.072	1.033	0.186
cFDA800_1	275	Fluconazole	L12	1.001	0.061	0.955	0.055
cFDA800_2	416	Azacitidine	D8	1.001	0.038	0.814	0.126
cFDA800_2	439	Capreomycin Sulfate	F14	1.001	0.038	1.016	0.037
cFDA800_3	641	Methyl Aminolevulinate·HCl	B17	1.001	0.022	1.062	0.184
cFDA800_3	726	Reserpine	C8	1.001	0.034	1.074	0.268
cFDA800_1	33	Pindolol	D7	1.002	0.042	0.878	0.022

cFDA800_3	315	Oxcarbazepine	L12	1.002	0.028	0.979	0.156
cFDA800_3	731	Rizatriptan Benzoate	C18	1.002	0.017	0.992	0.14
cFDA800_3	737	Selegiline·HCl	D10	1.002	0.05	1.027	0.133
cFDA800_3	762	Tetrahydrozoline·HCl	F20	1.002	0.046	1.055	0.138
cFDA800_1	12	Loperamide·HCl	B5	1.003	0.03	1.097	0.074
cFDA800_1	51	Aminophylline	F3	1.003	0.042	1.004	0.091
cFDA800_1	93	Dorzolamide·HCl	B8	1.003	0.056	1.053	0.116
cFDA800_2	374	Tamoxifen Citrate	F9	1.003	0.021	0.942	0.031
cFDA800_2	552	Fexofenadine·HCl	I20	1.003	0.025	0.939	0.149
cFDA800_3	90	Aripiprazole	K22	1.004	0.013	1.052	0.15
cFDA800_1	138	Ibuprofen	F18	1.004	0.025	1.02	0.076
cFDA800_2	484	Colistin Sulfate	K3	1.004	0.024	0.981	0.114
cFDA800_3	721	Raltegravir	B18	1.004	0.067	1.074	0.076
cFDA800_3	752	Telbivudine	E20	1.004	0.057	1.013	0.182
cFDA800_1	121	Felodipine	E4	1.005	0.019	0.944	0.049
cFDA800_1	203	Pramipexole Dihydrochloride Monohydrate	M7	1.005	0.103	0.94	0.057
cFDA800_3	312	Nystatin	L6	1.005	0.009	1.047	0.162
cFDA800_2	334	Nelfinavir Mesylate	B9	1.005	0.035	0.998	0.018
cFDA800_2	358	Bupivacaine·HCl	D17	1.005	0.048	0.932	0.081
cFDA800_3	636	Methotrexate	B7	1.005	0.096	1.059	0.166
cFDA800_3	652	Milnacipran·HCl	C19	1.005	0.09	1.083	0.153
cFDA800_3	697	Phenelzine Sulfate	H9	1.005	0.057	1.008	0.238
cFDA800_1	38	Ipratropium·Br	D17	1.006	0.025	0.932	0.075
cFDA800_1	161	Lapatinib Ditosylate	I3	1.006	0.017	0.992	0.044
cFDA800_1	224	Vinblastine Sulfate	O9	1.006	0.085	0.867	0.026
cFDA800_1	284	Idoxuridine	M10	1.006	0.04	0.975	0.046
cFDA800_2	379	Fulvestrant	F19	1.006	0.046	0.93	0.059
cFDA800_2	414	Atomoxetine·HCl	D4	1.006	0.056	0.936	0.097
cFDA800_2	556	Fludarabine Phosphate	J8	1.006	0.009	1.014	0.021
cFDA800_3	661	Naftifine·HCl	D17	1.006	0.09	1.01	0.176
cFDA800_3	2	Felbamate	K5	1.007	0.066	0.91	0.196
cFDA800_1	152	Goserelin Acetate	H6	1.007	0.022	1.014	0.122
cFDA800_2	488	Cycloserine	K11	1.007	0.057	0.972	0.067
cFDA800_2	549	Everolimus	I14	1.007	0.032	0.966	0.022
cFDA800_3	735	Rufinamide	D6	1.007	0.112	0.976	0.164
cFDA800_1	24	Prazosin·HCl	C9	1.008	0.057	1.032	0.029
cFDA800_1	72	Isoniazid	H5	1.008	0.046	1.028	0.057
cFDA800_1	149	Terbinafine·HCl	G20	1.008	0.06	0.946	0.064
cFDA800_1	198	Lomustine	L17	1.008	0.01	0.953	0.08
cFDA800_3	316	Oxiconazole Nitrate	L14	1.008	0.026	1.119	0.111

cFDA800_2	343	Telmisartan	C7	1.008	0.06	1.006	0.04
cFDA800_2	449	Cefixime	G14	1.008	0.058	0.915	0.105
cFDA800_3	618	Mechlorethamine·HCl	N12	1.008	0.027	1.022	0.132
cFDA800_3	717	Pyridostigmine Bromide	B10	1.008	0.068	0.652	0.541
cFDA800_2	575	Halobetasol Propionate	L6	1.009	0.032	0.976	0.044
cFDA800_3	759	Terconazole	F14	1.009	0.041	0.984	0.064
cFDA800_1	92	Butenafine·HCl	B6	1.01	0.011	1.066	0.048
cFDA800_1	157	Delavirdine Mesylate	H16	1.01	0.045	0.983	0.031
cFDA800_2	518	Digoxin	N11	1.01	0.039	0.914	0.104
cFDA800_3	623	Mepivacaine·HCl	N22	1.01	0.059	1.035	0.235
cFDA800_3	667	Nevirapine	E9	1.01	0.041	1.105	0.234
cFDA800_1	23	Amantadine·HCl	C7	1.011	0.03	1.009	0.08
cFDA800_1	42	Haloperidol	E5	1.011	0.041	0.995	0.026
cFDA800_1	43	Cimetidine	E7	1.011	0.048	0.997	0.089
cFDA800_3	82	Olanzapine	K6	1.011	0.025	1.032	0.104
cFDA800_3	87	Atovaquone	K16	1.011	0.021	0.914	0.074
cFDA800_1	164	Ergotamine Tartrate	I9	1.011	0.007	1.033	0.03
cFDA800_2	380	Esmolol	F21	1.011	0.045	0.927	0.035
cFDA800_2	426	Benztropine Mesylate	E8	1.011	0.075	0.968	0.049
cFDA800_3	543	Ethinyl Estradiol	N21	1.011	0.037	0.603	0.52
cFDA800_1	62	Ranitidine·HCl	G5	1.012	0.042	0.998	0.07
cFDA800_1	252	Dextromethorphan	J6	1.012	0.029	1.048	0.142
cFDA800_2	558	Flunisolide	J12	1.012	0.035	1.021	0.08
cFDA800_3	649	Blank	C13	1.012	0.05	1.03	0.12
cFDA800_3	793	Vancomycin·HCl	I21	1.012	0.039	1.035	0.108
cFDA800_1	46	Naltrexone·HCl	E13	1.013	0.016	0.987	0.019
cFDA800_1	223	Atracurium Besylate	O7	1.013	0.064	0.927	0.004
cFDA800_2	361	Fluoxetine·HCl	E3	1.013	0.048	0.901	0.046
cFDA800_2	364	Thioridazine·HCl	E9	1.013	0.025	0.946	0.071
cFDA800_2	550	Ezetimibe	I16	1.013	0.025	0.992	0.059
cFDA800_2	564	Fluvoxamine Maleate	K4	1.013	0.012	0.979	0.004
cFDA800_1	55	Atropine Sulfate Monohydrate	F11	1.014	0.017	0.965	0.133
cFDA800_1	132	Capsaicin	F6	1.014	0.034	0.931	0.059
cFDA800_1	134	Dipyridamole	F10	1.014	0.033	0.941	0.051
cFDA800_1	179	Letrozole	J19	1.014	0.048	0.907	0.043
cFDA800_1	197	Imipenem	L15	1.014	0.064	0.919	0.034
cFDA800_1	254	Zalcitabine (2',3'-Dideoxycytidine)	J10	1.014	0.022	0.91	0.117
cFDA800_1	257	Doxazosin Mesylate	J16	1.014	0.048	0.929	0.035
cFDA800_2	382	Succinylcholine Chloride·2H2O	G5	1.014	0.066	0.943	0.03
cFDA800_2	471	Cinacalcet·HCl	I17	1.014	0.058	1.006	0.089

cFDA800_3	542	Blank	N19	1.014	0.036	0.998	0.126
cFDA800_3	669	Nicotine	E13	1.014	0.081	1.054	0.269
cFDA800_1	269	Fluorouracil (5-Fluorouracil)	K20	1.015	0.063	0.982	0.037
cFDA800_2	451	Cefoxitin·Na	G18	1.015	0.016	1.011	0.135
cFDA800_2	476	Clobazam	J7	1.015	0.004	0.944	0.058
cFDA800_3	537	Erythromycin	N9	1.015	0.106	0.996	0.026
cFDA800_2	563	Fluticasone Propionate	J22	1.015	0.017	1.031	0.045
cFDA800_2	568	Fosfomycin Calcium	K12	1.015	0.01	0.903	0.03
cFDA800_3	714	Propylthiouracil	B4	1.015	0.052	1.064	0.082
cFDA800_1	57	Chlorpromazine·HCl	F15	1.016	0.041	0.9	0.042
cFDA800_1	148	Meloxicam	G18	1.016	0.011	1.042	0.017
cFDA800_2	431	Bisoprolol Fumarate	E18	1.016	0.022	0.953	0.023
cFDA800_2	565	Fomepizole	K6	1.016	0.012	0.994	0.047
cFDA800_1	49	Riluzole·HCl	E19	1.017	0.029	0.951	0.124
cFDA800_1	56	Apomorphine·HCl Hemihydrate	F13	1.017	0.164	0.928	0.061
cFDA800_1	174	Tolazamide	J9	1.017	0.046	1.149	0.053
cFDA800_2	487	Cyclopentolate	K9	1.017	0.056	1.006	0.09
cFDA800_3	654	Mitotane	D3	1.017	0.019	1.075	0.063
cFDA800_3	4	Donepezil·HCl	K9	1.018	0.035	0.914	0.094
cFDA800_1	105	Montelukast·Na	C12	1.018	0.053	0.955	0.127
cFDA800_1	166	Valproic Acid	I13	1.018	0.019	0.974	0.047
cFDA800_1	221	Ampicillin Trihydrate	O3	1.018	0.095	1.029	0.06
cFDA800_3	388	Acetaminophen	M12	1.018	0.083	1.036	0.223
cFDA800_2	394	Adefovir Dipivoxil	B4	1.018	0.065	1.064	0.054
cFDA800_2	446	Cefazolin·Na	G8	1.018	0.046	0.94	0.088
cFDA800_2	553	Fingolimod	I22	1.018	0.07	0.94	0.05
cFDA800_3	638	Methscopolamine Bromide ((−)-Scopolamine Methyl Bromide)	B11	1.018	0.067	1.105	0.094
cFDA800_1	78	Olopatadine	H17	1.019	0.066	0.958	0.087
cFDA800_1	188	Fosinopril·Na	K17	1.019	0.034	0.896	0.106
cFDA800_2	372	Mifepristone	F5	1.019	0.037	0.952	0.042
cFDA800_2	377	Efavirenz	F15	1.019	0.049	0.95	0.166
cFDA800_2	467	Ciclesonide	I9	1.019	0.049	1.02	0.037
cFDA800_2	548	Etonogestrel	I12	1.019	0.019	0.98	0.08
cFDA800_1	113	Galantamine·HBr	D8	1.02	0.067	1.049	0.082
cFDA800_3	240	Citalopram·HBr	L21	1.02	0.038	1.029	0.222
cFDA800_1	265	Fenoldopam Mesylate	K12	1.02	0.024	0.951	0.111
cFDA800_1	286	Imiquimod	M14	1.02	0.053	0.988	0.042
cFDA800_3	393	Acrivastine	M22	1.02	0.072	1.025	0.138
cFDA800_2	508	Dexmedetomidine·HCl	M11	1.02	0.038	0.963	0.053
cFDA800_3	630	Metaraminol Bitartrate	O15	1.02	0.028	1.033	0.051

cFDA800_3	723	Rasagiline Mesylate	B22	1.02	0.09	1.116	0.139
cFDA800_3	795	Vigabatrin	J5	1.02	0.042	1.027	0.104
cFDA800_1	162	Pioglitazone·HCl	I5	1.021	0.004	1.022	0.033
cFDA800_1	172	Glyburide	J5	1.021	0.047	1.022	0.091
cFDA800_3	327	Prednisone	M15	1.021	0.07	1.04	0.156
cFDA800_2	373	Megestrol Acetate	F7	1.021	0.054	1.003	0.066
cFDA800_2	450	Cefotetan Disodium	G16	1.021	0.013	0.932	0.125
cFDA800_3	664	Nebivolol·HCl	E3	1.021	0.026	1.037	0.191
cFDA800_3	777	Travoprost	H10	1.021	0.025	1.083	0.112
cFDA800_3	798	Zaleplon	J11	1.021	0.072	1.077	0.174
cFDA800_1	115	Amlodipine	D12	1.022	0.027	0.934	0.108
cFDA800_2	489	Cysteamine·HCl	K13	1.022	0.03	0.942	0.064
cFDA800_2	585	Ibutilide Fumarate	M6	1.022	0.08	0.942	0.047
cFDA800_3	788	Trospium Chloride	I11	1.022	0.06	1.049	0.132
cFDA800_1	50	Propofol	E21	1.023	0.036	0.995	0.05
cFDA800_1	70	Carbachol (Carbamylcholine) Chloride	G21	1.023	0.025	1.008	0.054
cFDA800_1	73	Ticlopidine·HCl	H7	1.023	0.011	1.013	0.071
cFDA800_1	173	Minoxidil	J7	1.023	0.03	1.138	0.223
cFDA800_3	389	Acetazolamide	M14	1.023	0.037	0.703	0.58
cFDA800_2	447	Cefdinir	G10	1.023	0.022	0.959	0.052
cFDA800_2	591	Isosorbide Dinitrate	M18	1.023	0.024	0.999	0.112
cFDA800_3	678	Orlistat (Tetrahydrolipstatin)	F11	1.023	0.062	0.647	0.549
cFDA800_1	180	Anastrozole	J21	1.024	0.027	0.888	0.178
cFDA800_1	215	Alendronate·Na Trihydrate	N11	1.024	0.079	0.91	0.079
cFDA800_1	280	Gemfibrozil	L22	1.024	0.058	0.916	0.041
cFDA800_2	424	Bendamustine·HCl	E4	1.024	0.03	0.957	0.073
cFDA800_2	512	Diazepam	M19	1.024	0.017	0.925	0.07
cFDA800_3	629	Metaproterenol Hemisulfate (Orciprenaline)	O13	1.024	0.066	0.991	0.167
cFDA800_3	670	Nilotinib	E15	1.024	0.077	0.836	0.248
cFDA800_1	259	Enalapril	J20	1.025	0.037	0.934	0.113
cFDA800_3	325	Progesterone	M11	1.025	0.029	0.989	0.049
cFDA800_2	362	Ondansetron	E5	1.025	0.067	0.954	0.067
cFDA800_2	480	Clotrimazole	J15	1.025	0.045	0.94	0.085
cFDA800_3	644	Metyrapone	C3	1.025	0.038	1.047	0.042
cFDA800_1	29	Betaxolol·HCl	C19	1.026	0.051	0.942	0.037
cFDA800_1	58	Fluphenazine·HCl	F17	1.026	0.016	0.946	0.168
cFDA800_1	182	Clindamycin Palmitate·HCl	K5	1.026	0.045	0.966	0.083
cFDA800_1	304	Minocycline	O10	1.026	0.047	0.985	0.057
cFDA800_1	144	Piroxicam	G10	1.027	0.028	0.96	0.064
cFDA800_2	433	Bromfenac	E22	1.027	0.021	0.931	0.051

cFDA800_2	466	Chlorzoxazone	I7	1.027	0.048	0.992	0.101
cFDA800_2	561	Flurandrenolide	J18	1.027	0.035	0.948	0.074
cFDA800_3	666	Nepafenac	E7	1.027	0.06	1.006	0.092
cFDA800_3	8	Spectinomycin·HCl Pentahydrate	K17	1.028	0.026	1.104	0.285
cFDA800_2	432	Brimonidine	E20	1.028	0.053	0.903	0.038
cFDA800_3	785	Trimethadione	I5	1.028	0.031	0.959	0.27
cFDA800_1	271	Amitriptyline·HCl	L4	1.029	0.063	0.995	0.045
cFDA800_3	391	Acetohydroxamic Acid	M18	1.029	0.028	1.033	0.05
cFDA800_1	122	Phenoxybenzamine·HCl	E6	1.03	0.01	0.982	0.109
cFDA800_1	289	Levonorgestrel	M20	1.03	0.048	0.979	0.063
cFDA800_3	317	Oxacillin·Na	L16	1.03	0.044	1.115	0.211
cFDA800_2	456	Ceftriaxone·Na	H8	1.03	0.023	1.001	0.037
cFDA800_2	500	Deferoxamine Mesylate	L15	1.03	0.069	0.938	0.074
cFDA800_1	201	Oseltamivir Phosphate	M3	1.031	0.04	1.017	0.089
cFDA800_1	226	Aztreonam	O13	1.031	0.088	1.006	0.048
cFDA800_1	229	Buspirone·HCl	O19	1.031	0.081	0.907	0.055
cFDA800_1	247	Cyclophosphamide (Free Base)	I16	1.031	0.052	0.945	0.047
cFDA800_2	436	Bupropion	F8	1.031	0.08	0.957	0.027
cFDA800_1	287	Indapamide	M16	1.032	0.035	0.974	0.1
cFDA800_2	464	Chlorpropamide	I3	1.032	0.017	1.023	0.032
cFDA800_2	567	Foscarnet·Na (Sodium Phosphonoformate Tribasic Hexahydrate)	K10	1.032	0.042	1.031	0.094
cFDA800_1	15	Phentolamine·HCl	B11	1.033	0.04	1.103	0.133
cFDA800_1	75	Vardenafil	H11	1.033	0.055	1.012	0.092
cFDA800_2	544	Ethionamide	I4	1.033	0.003	1.034	0.124
cFDA800_2	586	Iloperidone	M8	1.033	0.028	1.049	0.062
cFDA800_3	616	Mannitol	N8	1.033	0.03	1.087	0.203
cFDA800_3	722	Ramelteon	B20	1.033	0.048	1.155	0.179
cFDA800_3	799	Zanamivir	J13	1.033	0.049	0.981	0.028
cFDA800_1	266	Fenoprofen Calcium	K14	1.034	0.099	0.958	0.024
cFDA800_1	277	Ganciclovir	L16	1.034	0.064	0.932	0.054
cFDA800_3	328	Primaquine Phosphate	M17	1.034	0.011	1.02	0.116
cFDA800_3	658	Mupirocin	D11	1.034	0.09	1.068	0.19
cFDA800_1	211	Acyclovir (Acycloguanosine) Zovirax	N3	1.035	0.087	1.025	0.038
cFDA800_3	235	Chlorambucil	L11	1.035	0.077	0.936	0.131
cFDA800_1	274	Flutamide	L10	1.035	0.105	1.004	0.035
cFDA800_3	719	Quinidine·HCl·H2O	B14	1.035	0.091	1.058	0.074
cFDA800_1	186	Enalaprilat Maleate	K13	1.036	0.033	0.931	0.186
cFDA800_1	187	Fluvastatin·Na	K15	1.036	0.072	0.795	0.047
cFDA800_1	255	Diflunisal	J12	1.036	0.071	1.018	0.035
cFDA800_2	514	Dicyclomine·HCl	N3	1.036	0.039	0.922	0.044

cFDA800_3	536	Eptifibatide	N7	1.036	0.053	0.985	0.018
cFDA800_3	237	Chloroquine Diphosphate	L15	1.037	0.063	1.019	0.122
cFDA800_3	314	Omeprazole	L10	1.037	0.07	1.108	0.072
cFDA800_2	551	Febuxostat	I18	1.037	0.006	0.987	0.1
cFDA800_3	730	Ritonavir	C16	1.037	0.135	1.006	0.235
cFDA800_1	190	Granisetron·HCl	K21	1.038	0.043	0.907	0.096
cFDA800_3	238	Thalidomide	L17	1.038	0.058	1.037	0.11
cFDA800_1	264	Famciclovir	K10	1.038	0.073	0.993	0.027
cFDA800_2	376	Bosentan	F13	1.038	0.027	0.946	0.043
cFDA800_2	493	Dapsone	K21	1.038	0.04	1.003	0.068
cFDA800_2	594	Kanamycin Sulfate	N4	1.038	0.057	1.02	0.064
cFDA800_1	65	Quetiapine Fumarate	G11	1.039	0.03	0.936	0.06
cFDA800_1	94	Escitalopram	B10	1.039	0.057	1.016	0.071
cFDA800_2	482	Colchicine	J19	1.039	0.036	1.03	0.18
cFDA800_3	539	Estropipate	N13	1.039	0.055	1.045	0.172
cFDA800_2	560	Fluorometholone	J16	1.039	0.042	0.993	0.054
cFDA800_3	668	Niacin (Known As Vitamin B3, Nicotinic Acid And Vitamin Pp)	E11	1.039	0.06	0.984	0.057
cFDA800_3	713	Proparacaine·HCl	O22	1.039	0.023	0.863	0.04
cFDA800_1	185	Dolasetron	K11	1.04	0.038	0.929	0.021
cFDA800_3	648	Miconazole	C11	1.04	0.108	0.987	0.125
cFDA800_2	578	Hydralazine·HCl	L12	1.041	0.081	0.947	0.005
cFDA800_2	605	Levocarnitine	O6	1.041	0.04	0.98	0.077
cFDA800_1	163	Rivastigmine Tartrate	I7	1.042	0.021	1.095	0.074
cFDA800_1	278	Gatifloxacin	L18	1.042	0.01	0.992	0.056
cFDA800_1	308	Naphazoline·HCl	O18	1.042	0.058	1.044	0.033
cFDA800_2	573	Guanidine·HCl	K22	1.042	0.088	0.975	0.056
cFDA800_2	582	Hydroxychloroquine Sulfate	L20	1.042	0.016	0.951	0.048
cFDA800_3	632	Methacholine Chloride	O19	1.042	0.048	0.845	0.312
cFDA800_2	496	Darunavir	L7	1.043	0.066	0.981	0.058
cFDA800_3	619	Meclizine Dihydrochloride	N14	1.043	0.062	1.042	0.122
cFDA800_1	281	Glimepiride	M4	1.044	0.056	1.057	0.045
cFDA800_1	299	Methyldopa Sesquihydrate (L-A-Methyl-Dopa Sesquihyrate)	N20	1.044	0.055	0.967	0.065
cFDA800_1	196	Ibandronate·Na Monohydrate	L13	1.045	0.014	0.984	0.053
cFDA800_3	236	Chlorpheniramine Maleate	L13	1.045	0.114	0.991	0.159
cFDA800_1	253	Diclofenac·Na Salt	J8	1.045	0.027	0.977	0.154
cFDA800_2	465	Chlorthalidone	I5	1.045	0.042	1.072	0.018
cFDA800_2	547	Etomidate	I10	1.045	0.038	0.86	0.057
cFDA800_1	195	Cabergoline	L11	1.046	0.052	1.04	0.041
cFDA800_3	386	Acarbose	M8	1.046	0.04	0.908	0.142
cFDA800_3	781	Triamterene	H18	1.046	0.071	0.917	0.297

cFDA800_1	296	Medroxyprogesterone Acetate	N14	1.047	0.041	0.922	0.057
cFDA800_2	474	Cladribine	J3	1.047	0.048	1.058	0.164
cFDA800_2	477	Clofazimine	J9	1.047	0.014	1.026	0.109
cFDA800_2	587	Indinavir	M10	1.047	0.062	1.029	0.096
cFDA800_3	647	Micafungin	C9	1.047	0.079	1.053	0.26
cFDA800_2	499	Deferasirox	L13	1.048	0.095	1.005	0.065
cFDA800_3	747	Sulfanilamide	E10	1.048	0.09	0.978	0.076
cFDA800_3	750	Tadalafil	E16	1.048	0.2	0.873	0.104
cFDA800_3	10	Nicardipine·HCl	K21	1.049	0.055	1.071	0.193
cFDA800_1	217	Sumatriptan Succinate	N15	1.049	0.047	0.937	0.084
cFDA800_3	329	Praziquantel	M19	1.049	0.03	1.047	0.157
cFDA800_2	513	Dicloxacillin·Na Salt Monohydrate	M21	1.049	0.044	0.936	0.015
cFDA800_2	572	Griseofulvin	K20	1.049	0.045	0.866	0.039
cFDA800_3	701	Pitavastatin Calcium	H17	1.049	0.082	0.836	0.267
cFDA800_1	146	Carbidopa	G14	1.05	0.038	1.006	0.093
cFDA800_1	204	Triptorelin Acetate	M9	1.05	0.03	0.916	0.019
cFDA800_3	756	Teniposide	F8	1.05	0.048	0.989	0.231
cFDA800_3	318	Pantoprazole	L18	1.051	0.059	1.04	0.188
cFDA800_2	555	Flucytosine	J6	1.051	0.052	0.949	0.024
cFDA800_1	262	Estrone	K6	1.052	0.049	1.015	0.024
cFDA800_2	427	Betaine	E10	1.052	0.05	0.939	0.091
cFDA800_2	460	Chenodiol (Chenodeoxycholic Acid)	H16	1.052	0.059	1.032	0.104
cFDA800_2	517	Difluprednate	N9	1.052	0.056	1.016	0.06
cFDA800_2	520	Disopyramide	N15	1.052	0.003	0.917	0.047
cFDA800_1	100	Carvedilol	B22	1.053	0.066	1.062	0.098
cFDA800_1	210	Aspirin (Acetylsalicylic Acid)	M21	1.053	0.009	1.011	0.091
cFDA800_1	219	4-Aminosalicylic Acid	N19	1.053	0.116	0.961	0.055
cFDA800_2	491	Dalfampridine (4-Aminopyridine)	K17	1.053	0.032	0.968	0.052
cFDA800_2	528	Dutasteride	O11	1.053	0.008	1	0.026
cFDA800_2	569	Fosphenytoin·Na Pentahydrate	K14	1.053	0.018	1.009	0.043
cFDA800_2	607	Levothyroxine·Na	O10	1.053	0.06	1.008	0.086
cFDA800_3	706	Prasugrel	O8	1.053	0.037	1.06	0.109
cFDA800_1	206	Rocuronium Bromide	M13	1.054	0.057	0.971	0.085
cFDA800_1	302	Methimazole	O6	1.054	0.042	1.044	0.085
cFDA800_2	546	Etodolac	I8	1.054	0.031	0.979	0.054
cFDA800_1	270	Flurbiprofen	K22	1.055	0.007	0.905	0.085
cFDA800_3	330	Quinapril·HCl	M21	1.055	0.03	1.115	0.148
cFDA800_2	355	Valacyclovir·HCl	D11	1.055	0.064	0.846	0.06
cFDA800_2	554	Flavoxate·HCl	J4	1.055	0.042	1.017	0.072
cFDA800_1	272	Floxuridine	L6	1.056	0.071	0.988	0.047

cFDA800_2	479	Clonazepam	J13	1.056	0.015	0.949	0.078
cFDA800_2	557	Fludrocortisone Acetate	J10	1.056	0.026	0.949	0.046
cFDA800_2	562	Blank	J20	1.056	0.075	1.057	0.078
cFDA800_2	600	Lansoprazole	N16	1.056	0.053	0.963	0.055
cFDA800_2	580	Hydroflumethiazide	L16	1.057	0.015	0.984	0.134
cFDA800_2	584	Hydroxyzine Dihydrochloride	M4	1.057	0.051	1.011	0.038
cFDA800_3	685	Palonosetron·HCl	G5	1.057	0.021	0.865	0.114
cFDA800_1	126	Bromocriptine Mesylate	E14	1.058	0.036	0.945	0.023
cFDA800_1	192	Atazanavir	L5	1.058	0.07	0.969	0.069
cFDA800_2	335	Rimantadine·HCl	B11	1.058	0.042	1.02	0.066
cFDA800_2	470	Cilostazol	I15	1.058	0.009	1.055	0.012
cFDA800_2	583	Hydroxyurea	L22	1.058	0.051	0.925	0.062
cFDA800_3	712	Blank	O20	1.058	0.098	1.006	0.075
cFDA800_1	303	Metronidazole	O8	1.059	0.043	1.019	0.07
cFDA800_2	452	Cefpodoxime Proxetil	G20	1.059	0.087	0.933	0.083
cFDA800_2	502	Desipramine·HCl	L19	1.061	0.047	0.956	0.059
cFDA800_3	534	Epirubicin·HCl	N3	1.061	0.077	1.02	0.246
cFDA800_2	603	Levalbuterol·HCl	N22	1.061	0.056	0.958	0.113
cFDA800_1	54	Acetylcholine Chloride	F9	1.062	0.044	0.996	0.076
cFDA800_1	292	Lisinopril·2H2O	N6	1.063	0.108	0.959	0.058
cFDA800_2	468	Ciclopirox	I11	1.063	0.016	0.919	0.073
cFDA800_1	297	Mefenamic Acid	N16	1.064	0.114	0.925	0.078
cFDA800_3	392	Acetylcysteine	M20	1.064	0.06	1.05	0.107
cFDA800_2	574	Halcinonide	L4	1.064	0.029	0.919	0.165
cFDA800_2	611	Lorazepam	O18	1.064	0.079	0.972	0.067
cFDA800_2	613	Loxapine Succinate	O22	1.064	0.042	1.03	0.082
cFDA800_2	478	Clomipramine·HCl	J11	1.065	0.046	1.13	0.13
cFDA800_2	495	Darifenacin·HBr	L5	1.065	0.044	0.97	0.122
cFDA800_3	232	Cefotaxime Acid	L5	1.066	0.181	1.027	0.133
cFDA800_2	596	Labetalol·HCl	N8	1.066	0.039	0.919	0.075
cFDA800_3	624	Meprobamate (Schedule Iv)	O3	1.066	0.047	1.065	0.101
cFDA800_3	89	Cefepime·HCl Hydrate	K20	1.067	0.024	1.026	0.174
cFDA800_1	220	Mesalamine (5-Aminosalicylic Acid)	N21	1.067	0.049	0.934	0.043
cFDA800_3	239	Ciprofloxacin	L19	1.067	0.015	0.964	0.036
cFDA800_1	294	Losartan Potassium	N10	1.067	0.142	1.007	0.12
cFDA800_2	498	Decitabine	L11	1.067	0.065	1.008	0.028
cFDA800_2	533	Epinastine·HCl	O21	1.067	0.055	0.923	0.128
cFDA800_1	263	Etidronate Disodium	K8	1.068	0.012	1.042	0.074
cFDA800_1	279	Gentamycin Sulfate	L20	1.068	0.053	0.967	0.071
cFDA800_2	485	Cortisone Acetate	K5	1.068	0.084	1.017	0.032

cFDA800_2	522	Doripenem	N19	1.068	0.052	1.008	0.074
cFDA800_3	541	Ethambutol Dihydrochloride	N17	1.068	0.024	0.967	0.153
cFDA800_1	200	Meropenem	L21	1.071	0.106	0.92	0.033
cFDA800_1	306	Paclitaxel (Taxol)	O14	1.071	0.054	0.982	0.036
cFDA800_3	313	Ofloxacin	L8	1.071	0.101	1	0.112
cFDA800_3	707	Pravastatin·Na	O10	1.071	0.07	0.671	0.55
cFDA800_3	390	Acetohexamide	M16	1.072	0.029	1.144	0.213
cFDA800_2	521	Dopamine·HCl	N17	1.072	0.04	0.932	0.092
cFDA800_1	295	Mebendazole	N12	1.073	0.09	0.926	0.012
cFDA800_1	309	Nefazodone·HCl	O20	1.073	0.087	1.085	0.085
cFDA800_2	592	Isotretinoin (13-Cis-Retinoic Acid)	M20	1.073	0.046	0.925	0.058
cFDA800_2	599	Lamivudine	N14	1.073	0.041	0.951	0.042
cFDA800_3	620	Meclofenamate·Na	N16	1.073	0.02	0.872	0.32
cFDA800_3	621	Mefloquine·HCl	N18	1.073	0.022	1.062	0.113
cFDA800_3	705	Pralidoxime Chloride	O6	1.073	0.054	1.061	0.173
cFDA800_1	205	Risredonic Acid	M11	1.074	0.065	0.924	0.016
cFDA800_2	497	Dasatinib	L9	1.074	0.029	0.928	0.04
cFDA800_3	708	Pregabalin	O12	1.074	0.029	0.797	0.396
cFDA800_2	609	Liothyronine·Na	O14	1.075	0.081	0.924	0.076
cFDA800_1	285	Ifosfamide	M12	1.076	0.045	1.005	0.063
cFDA800_2	527	Duloxetine·HCl	O9	1.076	0.102	0.962	0.078
cFDA800_3	538	Estramustine Phosphate·Na	N11	1.076	0.028	1.023	0.123
cFDA800_3	736	Saquinavir Mesylate	D8	1.076	0.062	1.138	0.166
cFDA800_1	103	Imatinib Mesylate	C8	1.077	0.114	0.96	0.072
cFDA800_2	612	Loteprednol Etabonate	O20	1.077	0.106	1.018	0.134
cFDA800_1	214	Altretamine	N9	1.078	0.086	0.93	0.03
cFDA800_2	524	Doxepin·HCl	O3	1.078	0.056	1.001	0.048
cFDA800_3	617	Maraviroc	N10	1.078	0.057	1.015	0.135
cFDA800_3	711	Probenecid	O18	1.078	0.051	0.952	0.264
cFDA800_1	276	Furosemide	L14	1.079	0.062	0.954	0.039
cFDA800_2	506	Desvenlafaxine Succinate Hydrate	M7	1.079	0.044	0.954	0.061
cFDA800_3	323	Piperacillin	M7	1.08	0.072	0.983	0.185
cFDA800_2	589	Irinotecan·HCl	M14	1.08	0.05	0.876	0.044
cFDA800_2	597	Lacosamide	N10	1.08	0.038	0.95	0.075
cFDA800_3	710	Primidone	O16	1.08	0.01	0.777	0.429
cFDA800_1	298	Melphalan	N18	1.081	0.082	0.924	0.066
cFDA800_2	590	Isocarboxazid	M16	1.081	0.026	1.04	0.048
cFDA800_3	326	Procarbazine·HCl	M13	1.082	0.025	1.063	0.074
cFDA800_2	503	Desogestrel	L21	1.083	0.045	0.877	0.069
cFDA800_1	290	Levofloxacin·HCl	M22	1.084	0.047	0.987	0.031

cFDA800_1	213	Allopurinol	N7	1.085	0.021	0.925	0.051
cFDA800_2	570	Gemifloxacin	K16	1.085	0.056	0.945	0.123
cFDA800_2	602	Leucovorin Calcium Pentahydrate	N20	1.085	0.047	1.041	0.023
cFDA800_1	136	Indomethacin	F14	1.086	0.093	0.903	0.071
cFDA800_1	222	(±)-Atenolol	O5	1.086	0.107	0.937	0.078
cFDA800_2	523	Doxapram·HCl	N21	1.086	0.031	0.966	0.056
cFDA800_2	593	Isradipine	M22	1.086	0.088	0.974	0.058
cFDA800_2	579	Hydrochlorothiazide	L14	1.087	0.106	0.99	0.017
cFDA800_3	628	Mestranol	O11	1.087	0.076	1.099	0.091
cFDA800_2	417	Azelaic Acid	D10	1.088	0.014	0.943	0.101
cFDA800_2	494	Daptomycin	L3	1.088	0.028	1.084	0.03
cFDA800_2	504	Desonide	M3	1.088	0.037	0.985	0.079
cFDA800_2	531	Blank	O17	1.088	0.064	1.008	0.106
cFDA800_1	202	Pamidronate Disodium Pentahydrate (Pamidronic Acid)	M5	1.089	0.055	0.913	0.032
cFDA800_3	626	Mercaptopurine Hydrate	O7	1.089	0.038	0.846	0.21
cFDA800_2	516	Blank	N7	1.092	0.039	0.88	0.052
cFDA800_2	507	Dexchlorpheniramine Maleate	M9	1.093	0.048	0.971	0.07
cFDA800_2	604	Levobunolol·HCl	O4	1.093	0.058	1.048	0.015
cFDA800_2	610	Lopinavir	O16	1.093	0.091	1.044	0.073
cFDA800_3	633	Methazolamide	O21	1.093	0.058	1.034	0.104
cFDA800_1	310	Norethindrone	O22	1.094	0.057	0.973	0.04
cFDA800_3	631	Metaxalone	O17	1.094	0.017	1.105	0.247
cFDA800_2	519	Dimenhydrinate	N13	1.095	0.014	1.028	0.12
cFDA800_1	301	Metoprolol Tartrate	O4	1.096	0.114	0.931	0.04
cFDA800_2	601	Lenalidomide	N18	1.097	0.034	0.885	0.154
cFDA800_1	225	Azithromycin	O11	1.1	0.098	0.931	0.028
cFDA800_2	509	Blank	M13	1.1	0.053	0.971	0.041
cFDA800_2	510	Dexrazoxane	M15	1.1	0.05	0.959	0.03
cFDA800_2	515	Dienogest	N5	1.1	0.047	0.982	0.121
cFDA800_3	627	Mesna	O9	1.1	0.064	0.882	0.064
cFDA800_2	532	Eflornithine·HCl	O19	1.102	0.106	0.901	0.06
cFDA800_1	230	Carboplatin	O21	1.104	0.059	0.934	0.007
cFDA800_2	606	Levocetirizine Dihydrochloride	O8	1.104	0.088	0.952	0.069
cFDA800_3	622	Mepenzolate Bromide	N20	1.104	0.073	1.061	0.07
cFDA800_2	529	Dyphylline	O13	1.105	0.042	0.997	0.082
cFDA800_1	293	Loratadine	N8	1.106	0.053	0.99	0.023
cFDA800_1	216	Albendazole	N13	1.107	0.096	0.916	0.074
cFDA800_2	588	Irbesartan	M12	1.107	0.018	0.957	0.077
cFDA800_1	191	Oxaliplatin	L3	1.108	0.071	1.013	0.033
cFDA800_2	526	Drospirenone	O7	1.109	0.052	1.014	0.083

cFDA800_1	212	Zidovudine (3'-Azido-3'-Deoxythymidine)	N5	1.11	0.119	0.966	0.069
cFDA800_2	608	Lindane	O12	1.11	0.082	0.985	0.114
cFDA800_1	288	Itraconazole	M18	1.113	0.086	0.956	0.055
cFDA800_1	307	Nabumetone	O16	1.121	0.078	1.048	0.063
cFDA800_2	598	Lactulose	N12	1.121	0.047	0.93	0.092
cFDA800_1	291	Leflunomide	N4	1.126	0.074	1.08	0.062
cFDA800_3	709	Prilocaine·HCl	O14	1.126	0.058	1.061	0.049
cFDA800_3	704	Posaconazole	O4	1.178	0.133	1.018	0.118
cFDA800_2	430	Biperiden·HCl	E16	1.43	0.071	0.317	0.032
cFDA800_1	159	Doxorubicin·HCl	H20	2.675	0.496	0.735	0.105
cFDA800_1	158	Daunorubicin·HCl	H18	3.832	0.249	0.599	0.009
